# The Emerging Frontiers and Applications of High-Resolution 3D Printing

**DOI:** 10.3390/mi8040113

**Published:** 2017-04-01

**Authors:** Mao Mao, Jiankang He, Xiao Li, Bing Zhang, Qi Lei, Yaxiong Liu, Dichen Li

**Affiliations:** 1 State Key Laboratory for Manufacturing Systems Engineering, Xi’an Jiaotong University, Xi’an 710049, China; maomaosjs@outlook.com (M.M.); zhangbing_xjtu@163.com (B.Z.); leiqi310@stu.xjtu.edu.cn (Q.L.); yaxiongliu@mail.xjtu.edu.cn (Y.L.); dcli@mail.xjtu.edu.cn (D.L.); 2 Department of Chemistry, Stanford University, Stanford, CA 94305, USA; xil@stanford.edu

**Keywords:** high-resolution 3D printing, micro/nanofabrication, projection microstereoLithography, electrohydrodynamic printing, two-photon polymerization, direct ink writing

## Abstract

Over the past few decades, there has been an increasing interest in the fabrication of complex high-resolution three-dimensional (3D) architectures at micro/nanoscale. These architectures can be obtained through conventional microfabrication methods including photolithography, electron-beam lithography, femtosecond laser lithography, nanoimprint lithography, etc. However, the applications of these fabrication methods are limited by their high costs, the generation of various chemical wastes, and their insufficient ability to create high-aspect-ratio 3D structures. High-resolution 3D printing has recently emerged as a promising solution, as it is capable of building multifunctional 3D constructs with optimal properties. Here we present a review on the principles and the recent advances of high-resolution 3D printing techniques, including two-photon polymerization (TPP), projection microstereoLithography (PµSL), direct ink writing (DIW) and electrohydrodynamic printing (EHDP). We also highlight their typical applications in various fields such as metamaterials, energy storage, flexible electronics, microscale tissue engineering scaffolds and organ-on-chips. Finally, we discuss the challenge and perspective of these high-resolution 3D printing techniques in technical and application aspects. We believe that high-resolution 3D printing will eventually revolutionize the microfabrication processes of 3D architectures with high product quality and diversified materials. It will also find applications in a wide scope.

## 1. Introduction

There are growing demands for the fabrication of complex micro/nanoscale three-dimensional structures in various areas including novel materials, electronics, biomedical engineering, micro fuel cell, and microfluidics [1]. In the past, some technical approaches (e.g., electron-beam lithography, femtosecond laser lithography and nanoimprint lithography) have been broadly utilized to pattern materials with high-aspect-ratio structures. For example, inductively coupled plasma etching (ICP) was used to achieve batch fabrication of a large volume of silicon micropost array masters and mass-production of the elastomeric polydimethylsiloxane (PDMS) micropost arrays, which were used to study the effect of substrate rigidity on cell behavior and function [2]. Lithography in combination with thin film technologies was utilized to realize interconnects and micovias between device layers [3]. However, these techniques can only create 2D or 2.5D structures on a flat surface, which limits their applications. Moreover, they encounter challenges such as expensive equipment, complicated processing steps, long production period and the generation of various chemical wastes. In contrast to these subtractive manufacturing approaches that remove material, three-dimensional (3D) printing has emerged as a popular additive manufacturing technique to fabricate 3D geometries in a layer-by-layer manner, which achieves both macroscale customized architectures and complex internal structure. Currently, the resolution of conventional 3D printing techniques such as fused deposition modeling (FDM), stereolithography (SLA) and selective laser melting (SLM) is over 100 μm, which is insufficient to meet the demands for micro/nanoscale 3D structures.

High-resolution 3D printing techniques, which open new avenues for the generation of multifunctional 3D geometries with optimized properties, have quickly attracted the attention of researchers and been studied in various areas. These high-resolution 3D printing techniques are mainly based on photo-polymerization reactions, microextrution and micro-laser-sintering [3,4,5,6]. For example, two-photon polymerization of photosensitive materials has emerged as an effective approach for rapid and flexible fabrication of fully 3D structures with resolution as high as sub-100-nm [4]. 3D woodpile structures can be printed by direct writing with a rod diameter of 1 μm, which is defined by the diameter of microcapillary nozzles [6]. Electrohydrodynamic printing, which employs an electric field to reduce droplet size from a nozzle, is able to print complex 3D structures with high resolution (30 nm) using multiple functional inks [3]. 3D miniaturized systems such as metamaterials, biosensors and lab-on-chips have been manufactured through these techniques, showing numerous benefits of fabrication convenience, reliability, cost-effectiveness, and diversity of materials [7,8,9]. 

In this review, we summarize the recent progress of novel high-resolution 3D printing techniques including TPP, PµSL, DIW and EHDP. The working principles, fabrication resolution, merits and drawbacks of each technique are described. We then review their typical applications in various areas including novel materials, electronics and biomedicine. Here, we define “high-resolution 3D printing” as the technique that can fabricate structures with feature size smaller than 100 μm. However, the range of “high-resolution” for bioprinting is not so strict, since the feature size as large as ~200 μm is still sufficient for the construction of 3D patterned tissues, which has been difficult to achieve in the past.

## 2. High-Resolution 3D Printing Techniques 

### 2.1. Two-Photon Polymerization (TPP) 

**Figure 1:**
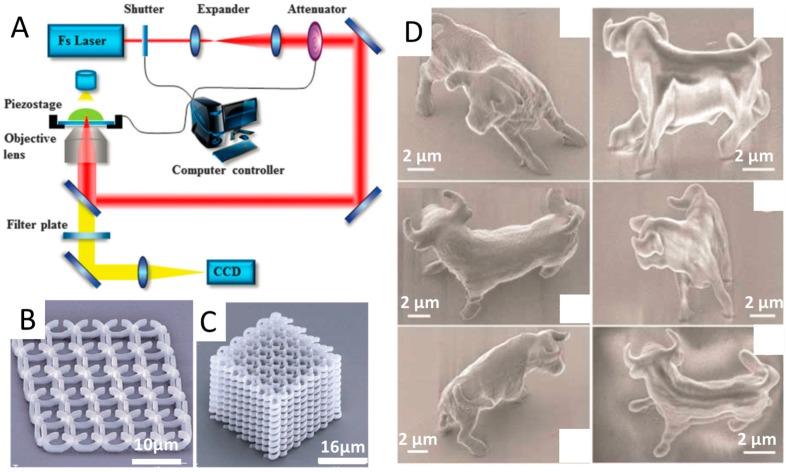
(**A**) Schematic of TPP, reprinted from [12] with permission of Royal Society of Chemistry, Copyright 2014. (**B**) 3D free-standing split-ring metamaterial structure; and (**C**) spiral photonic crystal fabricated using sol–gel material. (**B**,**C**) are reprinted from [4] with permission of Nature Publishing Group, Copyright 2009. (**D**) Bull sculptures produced by TPP at subdiffraction-limit resolution, reprinted from [13] with permission of Nature Publishing Group, Copyright 2001.

TPP has emerged as a promising 3D micro/nanoscale manufacturing tool for rapid and flexible fabrication of arbitrary and ultraprecise 3D structures with sub-100-nm resolution, which offers great opportunities to such fields as micro/nanophotonics, microfluidics and biomedical implants [10,11]. In a standard TPP system, there is a single femtosecond-pulsed laser beam (as shown in Figure 1A) [12]. The photopolymerization reaction requires the simultaneous absorption of two low-energy (typically near-IR) photons by a photoinitiator, thus the reaction kinetics are dependent on the square of the laser intensity. In this manner, the reaction is effectively confined to the focal point. By moving the beam focus in a 3D manner throughout the transparent material solution, 3D complex micro/nanoscale structures can be fabricated according to predesigned CAD models [4,13]. In contrast to conventional SLA, TPP can be considered as a true 3D printing technique without the need of supporting materials or layer-by-layer process, since the polymerization can happen within any 3D spatial position of a formulation, not limited to the surface. Figure 1B shows the 3D free-standing split-ring metamaterial structure fabricated by TPP method [4]. Moreover, in contrast to short wavelength ultraviolet (UV) light, which can induce photochemical damage to biological tissues, TPP utilizes the light of near-infrared (NIR) spectral range, making it a suitable photopolymerization process in presence of cells. The ability to produce arbitrary 3D structures with high resolution make TPP appealing for the fabrication of drug-delivery devices, implantable microelectromechanical systems (MEMS) and scaffolds for tissue engineering [11,12].

Since TPP was firstly demonstrated by Maruo and Kawata in the 1990s, notable progress has been made in improving the resolution of TPP technique [14]. Kawata et al. [13] demonstrated that the diffraction limit can be exceeded by nonlinear effects to obtain spatial resolution of 120 nm. In that work, tiny animal models of 10-μm-long and 7-μm-high bulls (as shown in Figure 1D) with the same size of a red blood cell were fabricated, which indicated that such micromachines had potential to be transported throughout the human vasculatures to deliver drugs. Xing et al. [15] improved lateral spatial resolution of TPP to 80 nm by using a highly sensitive and efficient photoinitiator, which introduced a low threshold and short exposure time. Their work demonstrated that the development of novel sensitive initiators was effective way to improve the resolution of TPP. Li et al. [16] developed a novel physical strategy to improve the resolution of TPP down to 40 nm, by employing pulsed 800 nm light to initiate cross-linking in a photoresist and single-photon absorption of continuous-wave 800 nm light simultaneously to deactivate the photopolymerization. Owing to its high-resolution, TPP proved to be an ideal approach to fabricate periodic optical nanostructures which can modify the motion of photons in the same way that ionic lattices affect electrons in solid [17]. The potential applications of TPP in various areas are also identified, such as micro-prism and micro-lens arrays, diffractive optical elements, and metamaterials [4,18]. For example, Figure 1C shows a spiral photonic crystal fabricated through TPP method using a sol-gel material. 

Multimaterial printing can also been achieved via TPP to fabricate 3D constructs with arbitrary microstructures precisely. Wylie et al. [19] successfully controlled the spatial immobilization of multiple growth factors within 3D hydrogels via two-photon photochemistry. Skylar-Scott et al. [20] generated 3D scaffolds with high-resolution patterned geometries and simultaneously introduced 3D patterns of protein cues into the scaffolds.

In brief, TPP is a promising 3D printing technique to create fully 3D structures with resolution as high as 30 nm, which cannot be achieved by many other approaches [21]. Commercial TPP equipment and resins have been launched by some companies such as Nanoscribe and utilized in some research institutions. Many disadvantages of conventional 3D printing are demonstrated to be avoided, such as the need of supporting materials, high surface tension, oxygen inhibition and a rough surface (induced by layer-by-layer printing manner). However, this approach comes with several disadvantages. For example, TPP is limited in its ability to fabricate macroscale object even higher than 1 cm due to its inherently serial nature. The cost of femtosecond lasers, positioning systems and optics is very high. The choices of resins for TPP are limited due to the requirement of high transparency to laser beams, and the adding of ceramic or metal particles into the liquid resins would be impossible. Novel materials for TPP with good optical, mechanical and biomedical properties need to be further explored.

### 2.2. Projection Microstereolithography (PµSL)

**Figure 2:**
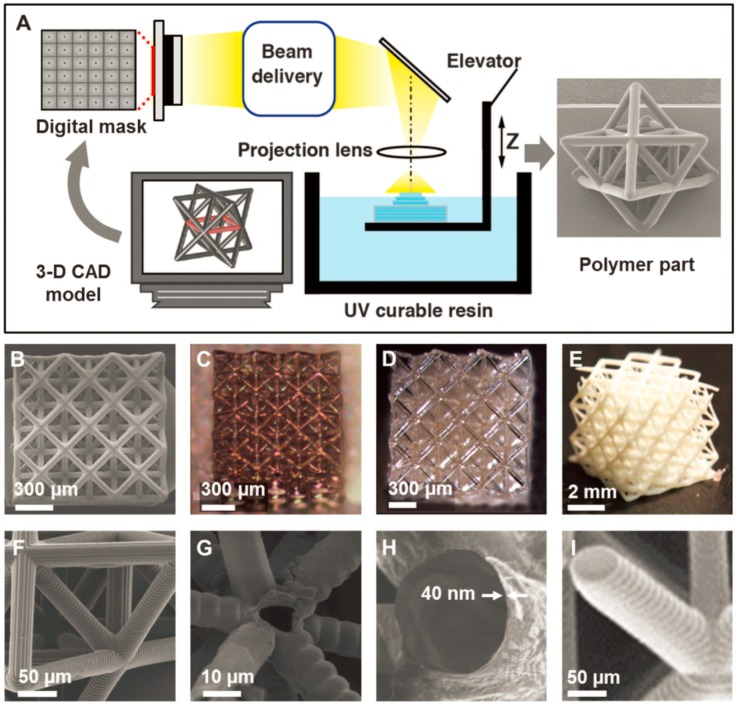
Fabrication of ultralight, ultrastiff mechanical microlattices: (**A**) fabrication process of projection microstereolithography; (**B**) solid polymer microlattices; (**C**) hollow-tube metallic microlattices; (**D**) hollow-tube ceramic microlattices; (**E**) solid ceramic microlattices; and (**F**–**I**) magnified views of the microlattices in (**B**–**E**), respectively. Reprinted from [24] with permission from the American Association for the Advancement of Science, Copyright 2014.

PµSL, a novel freeform high-throughput additive microfabrication technology, is capable of rapidly generating highly complex 3D microstructures with minimum feature size down to 1 μm in a layer-by-layer fashion [22]. Similar with conventional SLA method, PμSL is a 3D printing technique based on a photon-induced polymerization of liquid photosensitive materials (Figure 2A) [23,24,25]. The difference is that PµSL projects UV light through a mask to polymerize entire cross-sections in a single exposure, while conventional SLA method relies on the use of UV light to selectively solidify photosensitive materials by sequentially tracing 2D cross-sections of a 3D model. The submicron resolution of PµSL for the *x*, *y* and *z* translational stages is achieved by using high-quality digital mask with many micromirrors. To fabricate a 3D object through PµSL, an image corresponding to each layer is projected onto photosensitive materials, which will be polymerized to form the pattern as designed. Then, the polymerized layer is lowered into a resin bath and a new liquid resin layer will cover the top of the polymerized layer. This process is repeated until designed 3D object is completed. 

The development of PµSL is mainly focused on optimizing the projecting mask to improve the productivity. While the first generation of PµSL systems utilized physical glass masks for exposure, they were soon replaced by a digital dynamic mask, which allowed for modulating the multiple configured patterns without physically replacing the mask for each layer. In 1997, Bertsch et al. [26] utilized liquid crystal display (LCD) as the dynamic mask to obtain the designed pattern of each layer, reducing the cost and building time compared with glass masks. However, the large pixel size had limited the resolution of LCD-based PµSL. Sun et al. [22] successfully fabricate complex 3D microstructures(e.g., matrix, and micro-spring array) with the smallest feature of 0.6 μm through the PµSL technique using the Digital Micromirror Device (DMD, Texas Instruments, Dallas, TX, USA) as a dynamic mask. This DMD involves millions of micromirrors, each of which stands for 1 pixel in the projected pattern and can be controlled individually. The size and number of the mirrors determined the resolution of the projected images. 

In order to fabricate multi-scale 3D architected materials over a substantially larger size, a large-area projection microstereolithography is developed by combining scanning mechanism from laser-based stereolithography with the image projection optics of digital light processing (DLP) based stereolithography [5,27]. This is able to project the configured light pattern from the spatial light modulator onto the ultraviolet-light curable monomer surface, taking advantage of galvanometric mirrors combined with scanning lens. With a flat-field scanning lens and a fast scanning optics, 3D architected materials with microscale features can be fabricated with a building speed of 12,000 mm^3^/h and a large build plane of 100 cm^2^.

Hence, although the resolution is not as high as that of TPP technique, PµSL combines advantages of conventional SLA and projection lithography, allowing for high-throughput fabrication of complex 3D objects with microscale features. A variety of functional materials are available for PµSL such as polymers, shape memory polymers and biomaterials [28]. High-resolution metallic and ceramic microlattices can also be produced from the printed polymer parts, with nanoscale coating and postprocessing, as shown in Figure 2B–I. The relatively simple fast process and simple low-cost apparatus make PµSL a promising high-resolution 3D printing technique to be applied in more areas.

### 2.3. Direct Ink Writing (DIW)

**Figure 3:**
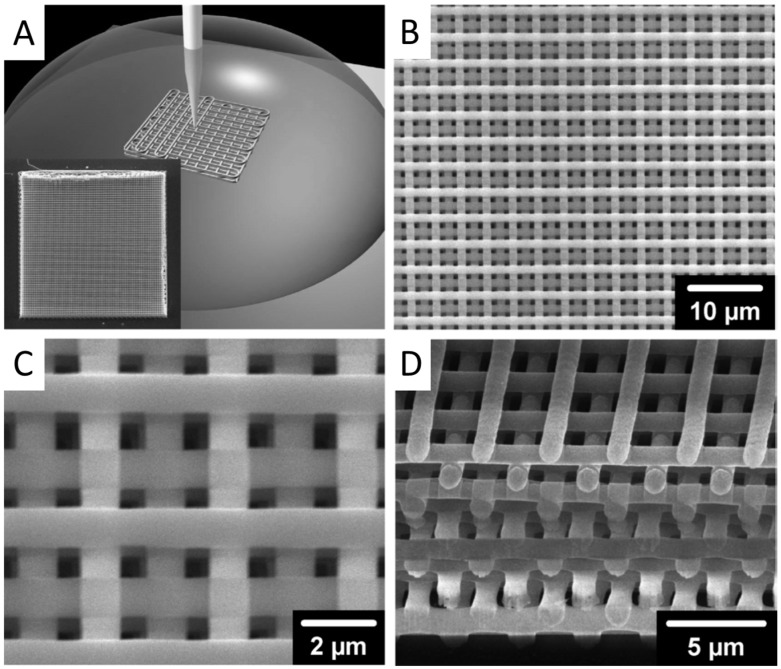
(**A**) Schematic illustration of direct ink writing (DIW); (**B**–**D**) woodpile structure fabricated by DIW: (**B**) intermediate-magnification top view; (**C**) high-magnification top view; (**D**) focused ion beam milled cross section. Reprinted from [29] with permission of John Wiley and Sons, Copyright 2006.

DIW includes a variety of 3D printing approaches that move an ink-depositing nozzle to create objects with controlled architectures and compositions predefined by CAD models. These inks solidify to form 3D objects when extruded under a pneumatic pressure either through liquid evaporation, gelation, or temperature- or solvent-induced phase change. The representative methods include filamentary-based approaches, such as fused deposition and droplet-based approaches, such as ink-jet printing. Usually, the resolution of DIW technique is defined by the diameter of printing nozzles. Ellis et al. [29] have proven that DIW could fabricate 3D lattices with minimum feature size down to 1 μm by using microcapillary nozzles of varying diameter (0.5–5.0 μm), as shown in Figure 3. 

One main advantage of direct ink writing is the diversity of printable materials, such as polymers, waxes, hydrogels, cell spheroids ceramics and even metals [8,30,31,32]. For example, a single elastomeric material was utilized as ink to print intricate spider webs directly according to computational modeling, to determine how web mechanics were controlled by their topological design and material distribution [33]. Skylar-Scott et al. [31] combined direct writing and laser sintering, which allowed for on-the-fly laser annealing during 3D printing. With this technology, they were able to make 3D free-standing metal structures, whose wire diameter could go down to <1 µm. Liquid metal (e.g., gallium alloys) is more recent 3D-printable conductive material [34]. DIW can also be easily used for multimaterial 3D printing with a high degree of spatial and compositional precision, to achieve the complexity and functional performance of printed objects. There are usually two types of ways for multimaterial DIW. One was achieved by using multiple printheads in a 3D printing system, each of which is filled with different ink composition. Kolesky et al. [35,36] implemented a custom-designed, large-area 3D bioprinter with four independently controlled printheads, each composed of fugitive or different cell-laden hydrogel inks, to produce thick tissue constructs. Vasculature and multiple cell types could be successfully placed within the printed 3D extracellular matrices in a programmable manner. Wehner et al. [37] used DIW method to fabricate an entirely soft, autonomous robot, named “octobot”, with eight arms that were powered by monopropellant decomposition through the programmable assembly of multiple materials. A new class of cardiac microphysiological devices instrumented with soft strain gauge sensors were successfully printed via DIW with six functional inks based on piezo-resistive, high-conductance, and bioavailable soft materials [38]. Another kind of multimaterial DIW is achieved by using microfluidic printheads that allow for switching, mixing or core-shell printing. Hardin et al. [39] demonstrated that multimaterial 3D printing using a microfluidic switching printhead could swap between two different viscoelastic inks seamlessly during the fabrication process as designed. The ability to print multiple materials from the same nozzle in a programmable manner opens a new avenue to generate functional 3D objects with predefined compositional and property gradients. Ober et al. [40] designed and implemented active mixing printheads for multimaterial 3D printing of viscoelastic inks that could efficiently homogenize a wide range of complex fluids at the microscale with tunable gradients of mechanical or conductive properties. Multicore–shell printing approach was utilized to fabricate capacitive soft strain sensors consisting of concentrically layered materials by developing a nonvolatile ionic fluid, modifying a soft silicone elastomer, and arranging them coaxially in a cylindrical capacitor motif [41]. These variants of DIW methods offer considerable flexibility in the fabrication of multiscale architectures with predefined composition distribution.

DIW has been demonstrated to be a high-resolution 3D printing technique to fabricate complex 3D architectures from a broad array of materials at a low cost. Its relative gentle printing process makes it a good choice for cell printing and many biological applications. However, the printing speed of DIW should be further improved, since the movement of nozzles is much slower than that of the light used in SLA or PµSL. Microfluidic multinozzle arrays were fabricated for high-throughput printing, which could potentially enhance the printing speed of DIW [42]. The most challenge for high-resolution DIW method lies in the material design and shear thinning to allow ink to be extruded under pressure/shear. Novel DIW printing systems should be developed to produce high-quality 3D components from functional materials, such as metals, ceramics, and smart materials, to achieve required thermal, mechanical, and electrical properties for industrial applications.

### 2.4. Electrohydrodynamic Printing (EHDP)

**Figure 4:**
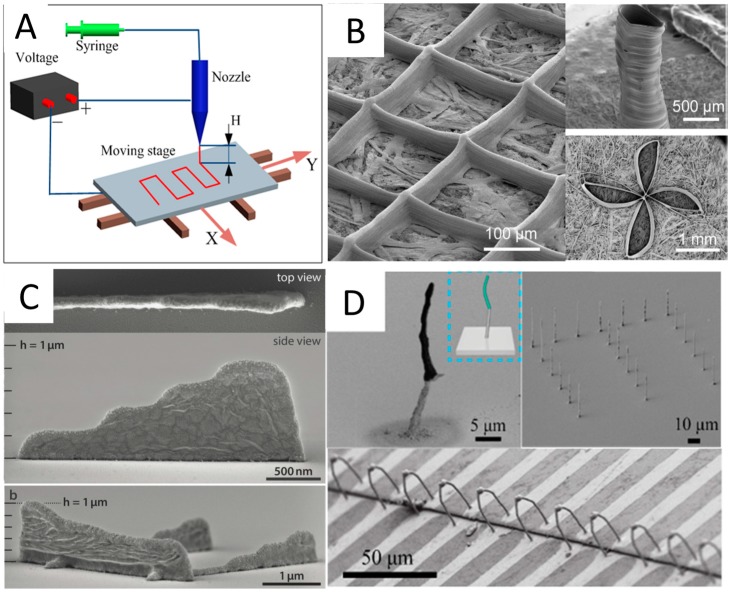
(**A**) The schematic diagram of electrohydrodynamic printing (EHDP), reprinted from [46] with permission of Royal Society of Chemistry, Copyright 2016. (**B**) Self-aligned electrospinning of 3D constructs through orderly fiber-by-fiber stacking with predesigned architectures, reprinted from [50] with permission of American Chemical Society, Copyright 2015. (**C**) Top and side views of electrohydrodynamic nanodrip printed nanowall with Width and maximal height of 120 and 870 nm, reprinted from [54] with permission of John Wiley and Sons, Copyright 2015. (**D**) Scanning electron microscopy (SEM) images of 3D freestanding structures printed through an electrohydrodynamic inkjet with multiple functional Inks, reprinted from [3] with permission of John Wiley and Sons, Copyright 2015.

EHDP, relying on electrified viscous fluid (solution or melt) jet, has gradually been considered to be a low-cost, reliable, high-throughput and high-resolution 3D printing technique to produce 3D objects with micro/nanoscale geometries using multiple functional inks [43,44]. During the EHDP process, electrically conductive viscous fluid from a pipette nozzle is subjected to an electrostatic field to form a Taylor-cone. If the electric stresses at the liquid–air interface ultimately overcome the resisting effect of surface tension, a fine jet or droplet is produced from the cone. The fine jet or droplet will be dried instantly with minimal solvent volumes before their arrival onto a moving collector and then stacked vertically at room temperature to produce 3D structures. Since the jet/droplet diameter is much smaller than the pipette nozzle size (500 nm to hundreds of microns) based on the principle of electrospinning, EHDP can print high-resolution 3D structures with 30 nm features. Typically, EHDP platforms consist of high voltage, syringe pump, jetting nozzle and controlled moving collector, as shown in Figure 4A. The high-resolution moving stage is really critical to define the print trajectory of 2D patterns or 3D structures. The applied voltage and the nozzle-to-collector distance determine the electric field strength, which can significantly affect electrohydrodynamic jetting mode as well as printed feature morphology [21,45]. There are also many other important factors for EHDP, including nozzle inner diameter, nozzle shape, stage moving speed, feeding rate and so on. Details can be found in review papers by Zhang et al. [46,47,48,49].

EHDP is becoming a feasible 3D process to fabricate micro/nanostructures in a layer-by-layer manner. Luo et al. [50] successfully addressed this issue and demonstrated a direct-write self-aligned 3D EHDP process by employing a printing paper placed on a grounded conductive collector. In this way, the paper absorbed redundant solvents and transferred residual charges to the ground, which subsequently promoted the precise deposition of printed fibers. High aspect ratio architectures such as grid, hollow cylinder and floral pattern were obtained as shown in Figure 4B. Lee et al. [51] found that the charged nanojet, which tended to become unstable when traveling in the air because of coulombic repulsion, could be stably focused onto the microline of a metal electrode and stack successively to fabricate nanoscale 3D objects. The diameter of nanojet used was as thin as 180 nm and 4.5-μm-tall, 220-μm-long nanowall was constructed. The percentages of polymer and solvent within printing inks were also optimized. Our group [52,53] developed EHDP platforms and verified its feasibility to print filaments with the diameter of about 10 μm, which could be precisely stacked into 3D tissue engineered scaffolds with complex curved geometries and a high-aspect-ratio of about 60. One type of EHDP approach in micro/nanodripping mode was investigated, which can print features sizes down to 30 nm [21]. By adjusting voltage frequency and stage moving speed, the ink can be ejected discretely, different from the continuous jetting way in common EHDP approaches as described above. Schneider et al. [54] combined the nanoscale resolution and the 3D capabilities of electrohydrodynamic nanodripping printing to fabricate high aspect ratio silver and gold metal grid transparent electrodes with the smallest feature size of 80 nm, as shown in Figure 4C. With a high aspect ratio, the electrical performance could be significantly increased while the overall optoelectronic performance still exceeded conventional ITO materials. Furthermore, An et al. [3] successfully printed various high-resolution 3D structures with multiple functional inks via EHDP process. Diverse materials, including organic light-emitting small molecules and metallic or magnetic nanoparticles were printed into relatively 3D complex shapes (e.g., walls, helical structures, and arch-like bridges) with resolutions of submicron scale. The freestanding Ag structures were successfully used for the connection of two separate electrodes, as demonstrated in Figure 4D.

It is envisioned that EHDP can provide an innovative way to fabricate multiple micro/nanoscale 3D structures in the foreseeable future. There are many remarkable advantages such as low cost, simple process control, high printing resolution and so on. However, there are still some great challenges to be addressed. For example, the problem of instability during the ejecting and stacking process should be overcome, which will affect the stacking accuracy and limit maximum height; the columbic repulsion caused by the residual charges will affect the stacking and bonding of printed layers; the speed of EHDP in micro/nanodripping mode are relatively slow. There is still a long way before EHDP definitely mature into a viable micro/nanoscale additive manufacturing technique for industrial applications.

## 3. Typical Applications of High-Resolution 3D Printing

### 3.1. Metamaterials

Metamaterials are engineered composites that exhibit exotic properties not found in nature and their constitute materials, such as subdiffraction confinement, electromagnetic cloaking and ultrahigh mechanical efficiency [24,55,56,57]. The special properties are derived from newly designed structures of the materials, which are usually arranged in repeating patterns such as unit cells [58,59,60]. Traditionally, these typical geometries are fabricated by top-down processes such as electron-beam lithography or focused-ion beam milling [61]. The self-assembly method of metallic colloids also provides a new down-top way for construction of complex 2D and 3D metamaterials [59,62], but structural controllability and suitable materials are still limited. Well known for its great manufacturing flexibility to create versatile objects with customizable shapes and programmable microarchitectures, high-resolution 3D printing was ideally a versatile and low-cost route to fabricate metamaterial with high precision and ultra-properties, which were used to study the critical connection between material microstructure, hierarchical architecture and material properties at relevant length scales [33,63,64]. 

**Figure 5:**
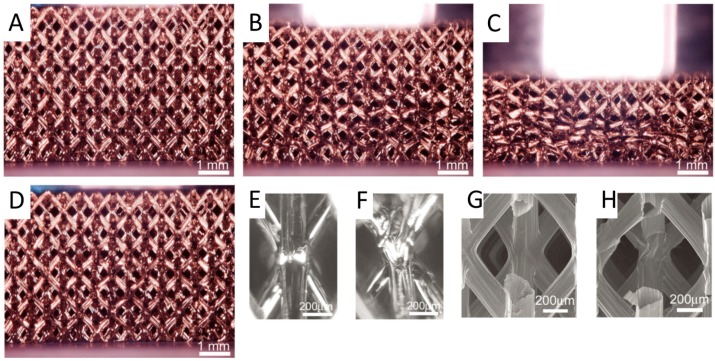
Ultralight metallic microlattices exhibit great characteristic of recoverable deformation: (**A**) before deformation; (**B**) 15% compression; (**C**) 50% compression; (**D**) full recovery after removal of load; (**E**) unit cell unloaded; (**F**) buckling node under compression; (**G**) SEM image of node before testing; and (**H**) SEM image of node after six compression cycles at 50% strain. Reprinted from [65] with the permission of American Association for the Advancement of Science, Copyright 2011.

In order to obtain “mechanical metamaterials” with super mechanical properties, high-resolution 3D printing techniques are employed to fabricate constructs mimicking many natural siliceous skeleton species that have ultra-mechanical properties due to their hierarchical design of components. Combined with a nanoscale coating method, 3D printing techniques-TPP and PµSL-are used to fabricate arbitrary 3D microarchitectures with controlled micro- and nanostructure across a wide range of mass density and material constitutes [27]. The fabrication process typical consists of the following steps: digital design of a three-dimensional structure, TPP or PµSL to create free-standing 3D solid polymer skeletons, conformal materials deposition using atomic layer deposition, and etching out of the polymer core to create hollow nanolattices. By following these steps, various brittle materials such as ceramics and metals, which are sensitive to flaws, could be transformed into strong, ultralight, energy-absorbing and recoverable metamaterials with 3D micro/nanoscale structures [9,64,65,66]. Zhu et al. [67] reported the fabrication of highly compressible 3D periodic graphene aerogel microlattices via DIW technique. The printed graphene aerogels were lightweight, highly conductive and exhibited supercompressibility up to 90% compressive strain. Using PμSL technique, Zheng et al. [27] fabricated multiscale metallic metamaterials with hybrid hierarchical topology, which were investigated to exhibit superelastic stretching deformation at the macroscale, a behavior not found in common bulk metal alloy or cellular metals. The combination of hybrid hierarchical architectures, distributed over successive hierarchies down to nanoscale thickness, contributed to the achievement of unprecedented scalability of high strength and ultralow density, as well as high compressive and superelastic tensile behavior. The printed metallic metamaterials were stretchable and compressible, with high tensile (~20%) and compressive elastic deformation (>50%) which were not observed in common lightweight metal foams or lattices [68,69]. Figure 5 demonstrates the great recoverable ability of ultralight metallic microlattices with 50% compression. 

Photonic metamaterials can manipulate light at optical frequencies, owing to their nanoscale structures which are smaller the wavelength of the visible spectrum. High-resolution 3D printing techniques such as TPP, EHDP and DIW have been demonstrated to be an effective pathway to fabricate these nanoscale structures. Hu et al. [70] used TPP to fabricate stable and actively tunable 3D photonic crystals with reversibly addressable refractive indices. The outstanding characteristics of printed photonic crystals such as switchability, high surface-to-volume ratio, and opportunities for further chemical functionalization of the intricate 3D surfaces, presented a range of opportunities in applications as displays and sensing devices. Yudistira et al. [71] successfully fabricated 3D multilayer terahertz metamaterial with a high refractive index of around 23.7 at large scale and with low cost, using EHDP technique as an alternative to the expensive conventional lithography method. Richner et al. [72] demonstrated that EHDP in a nanodripping mode was able to generate precise out-of-plane forests of nanoscopic metamaterial absorbers and images with diffraction-limited resolution on flat and nonflat substrates. The printed metamaterials consisted of composite nanopillars, which allowed the fine-tuning of the overall visible light absorption from complete absorption to complete reflection by simply tuning the pillar height. A nanopillar forest covering only 6% of the printed area could achieve 95% absorption of the entire visible spectrum. Gratson et al. [29] employed DIW technique to print 3D Si hollow-woodpile photonic crystals by combing direct-write assembly of concentrated polyelectrolyte inks with chemical vapor deposition of Si. The maximum observed reflectance of the final 3D Si hollow-woodpile was 0.3.

### 3.2. Energy Storage

**Figure 6:**
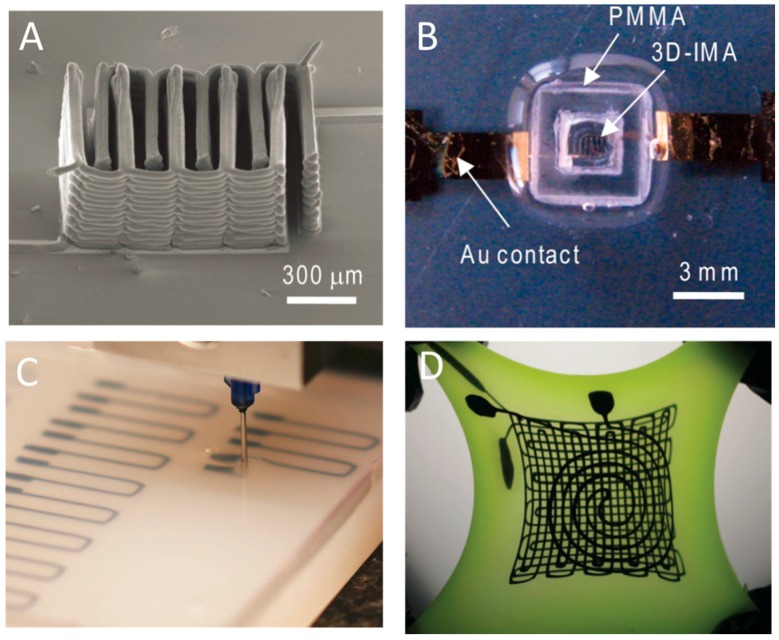
(**A**) SEM image of printed thin-walled Li_4_Ti_5_O_12_ (LTO)-LiFePO_4_ (LFP) electrode architectures; and (**B**) 3D LTO-LFP electrodes after packaging; (**A**,**B**) are reprinted from [73] with the permission of John Wiley and Sons, Copyright 2013. (**C**) Conductive ink was 3D printed inside uncured elastomeric reservoir; and (**D**) photograph of a three-layer strain sensor being stretched; (**C**,**D**) are reprinted from [7] with the permission of John Wiley and Sons, Copyright 2011.

Another typical application of high-resolution 3D printing is to fabricate micro/nano-architectures-based microbatteries for energy storage. Compared to conventional rechargeable batteries, 3D printed microbatteries could potentially double the energy density by fully utilizing limited space. For example, Sun et al. [73] printed novel 3D Li-ion microbatteries with the highest areal energy and power densities reported to date, using Li_4_Ti_5_O_12_ (LTO) as the anode material and LiFePO_4_ (LFP) as the cathode material. The composition and rheology of LFP and LTO viscoelastic ink materials were designed and optimized to ensure reliable flow through 3D printing nozzles with a diameter of 30 μm and promote adhesion between the printed features. Those were crucial to avoid delamination or distortion during drying and sintering, and achieve thin-walled electrode architectures, as shown in Figure 6A,B. However, due to the absence of conducting additives, their 3D printed Li-ion microbatteries still possessed low electrical conductivity about 10^−4^–10^−6^ S·cm^−1^, which significantly limited electrochemical performance of batteries. To improve the battery performance, Fu et al. [74] added graphene, an additive introducing good electrical conductivity, into cathode and anode materials (LEP and LTO) to make 3D printing inks with high viscosity and optimum viscoelastic properties. DIW-based 3D printing was then used to fabricate LFP cathode and LTO anode with complex 3D architectures, which exhibited stable cycling performance with specific capacities of ≈160 mAh·g^−1^ and ≈170 mA·g^−1^, respectively. Hu et al. [75] printed lithium-ion batteries with both ultrahigh rate and high capacity, using carbon coated LiMn_0.21_Fe_0.79_PO_4_ nanocrystals as printing ink to enhance electric conductivity. Moreover, they firstly demonstrated how to achieve ultrahigh rate performance for a cathode with LiMn_0.21_Fe_0.79_PO_4_ nanocrystals, by comparing theoretical Markov model with experimental data of both 3D-printed and traditional electrodes.

In another study, Sun et al. [76] employed DIW-based 3D printing technology to fabricate graphene-based planar micro-supercapacitors on flexible substrates layer-by-layer, using patterned laminated graphene films as interdigitated electrodes and polyvinyl alcohol-H_2_SO_4_ gel as the electrolyte. The resultant solid-state micro-supercapacitors were demonstrated to exhibit high volumetric energy and power density as well as good cycling stability, showing great potential for applications in microelectronics, lab-on-a-chip systems and flexible electronics. Zinc-silver has also been used to fabricate microbatteries because of its high energy and power densities [77]. For instance, arrays of pillars structure composed of alkaline zinc–silver were fabricated by ink-jet based 3D printing method, and the resultant pillared electrodes demonstrated an increase in capacity compared with planar electrodes of similar footprint area.

### 3.3. Flexible Electronics

Flexible electronics, representing a trending format of electronics, has been demonstrating their great potentials in areas such as information technology and medicine [78]. Evolved from the 2D printing technique for flexible electronics, 3D printable materials that are available with flexible substrates have been extending the functionalities and potential applications of flexible electronics [79]. 3D-printed electronics allow for 3D structural electronics which can stand alone or conform non-flat substrates [80,81,82,83].

Flexible electronics have been successfully printed by employing the DIW technique, with various proper inks developed. For example, Ahn et al. [84] printed stacked layers of concentrated ITO ink as microelectrodes. Muth et al. [7] inserted printing nozzles inside the uncured elastomeric reservoir, making it possible to embed 3D electronics inside a flexible substrate. Figure 6C,D demonstrates the printing process and the final 3D printed sensor in the stretched stage, respectively. Jiang et al. [85] adopted printing silver nanoparticle ink on top of liquid PDMS before sintering the ink and solidifying PDMS. The liquid PDMS confined the spreading of the printed ink, which generated very high conductive resolution line. Ladd et al. [34] printed a low viscosity liquid metal at room temperature into a variety of stable free-standing 3D microstructures, which could be incorporated on flexible substrates. They demonstrated 3D printing was a promising way to fabricate useful for soft, stretchable, or shape reconfigurable electronics. In another report, Wang et al. [86] printed graphene oxide nanoribbons to make fibers that could stack to form 3D lattices. The fabricated 3D integrated circuits are lighter and smaller than traditional metal circuits, with high electrical conductivity up to 680 S·cm^−1^. Kim et al. [87] proposed a DIW-based nanometer-scale 3D printing approach to write freestanding reduced graphene oxide nanowires using a size-controllable liquid meniscus. The 3D nanowires with diverse and complicated features can grow in any direction and at the selected sites, showing ideal properties for the production of components for flexible electronics.

To obtain flexible electronics with some functions beyond conventional electronic circuitry, functional conductive materials was mixed with 3D printable polymers. Leigh et al. [88] mixed polymorph thermoplastic and carbon black to prepare conductive printable filaments, which was then printed into conductive 3D structures. A test cube of this printed material had an in-plane resistivity of 0.09 ± 0.01 Ω·m^−1^ and perpendicular resistivity of 0.12 ± 0.01 Ω·m^−1^. More interestingly, the conductive filament might be printed alongside conventional 3D printed material to form structural electronics. In a recent report, Zhang et al. [89] mixed polylactic acid (PLA) with reduced graphene oxide, to make filaments, with which stretchable 3D conductive structures were made. The 3D printed composites obtained a very high conductivity of 600 S/cm, which might be contributed to the oriented microstructures of reduced graphene oxide produced by the printing process. 

### 3.4. Tissue Engineering Scaffolds

One of the most important applications of EHDP is to fabricate 3D tissue engineering scaffolds with controllable macroscopic geometries as well as microscale architectures. Various biopolymers have been used to build synthetic scaffolds via EHDP [90,91,92]. In consideration of the printed fibers possessing a suitable scale (about 800 nm–40 μm) which was very close to the scale of living cells, the synthetic scaffold could provide a biomimetic microenvironment for cellular attachment, proliferation and differentiation. 

**Figure 7:**
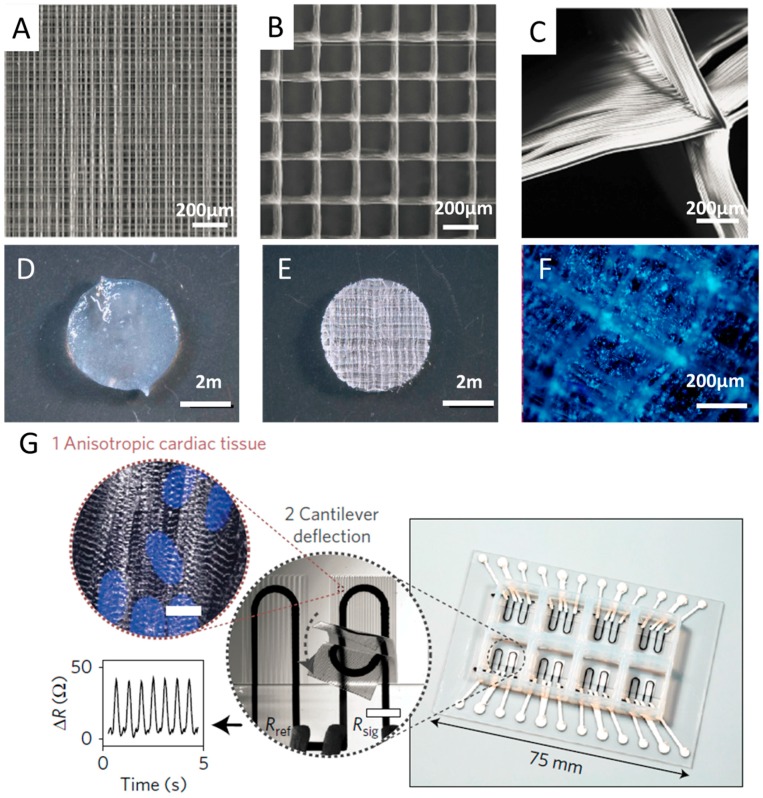
(**A**) 3D printed Polycaprolactone (PCL) scaffold with fibers spacing 0.2 mm; (**B**) 3D printed PCL scaffold with fibers spacing 1.0 mm; (**C**) detailed image of scaffold with fibers spacing 1.0 mm; (**D**) hydrogel without PCL scaffold; (**E**) hydrogel reinforced with a PCL scaffold; and (**F**) DAPI staining demonstrated the homogenous distribution of the cells throughout the construct; (**A**–**F**) are reprinted from [97] with permission of Nature Publishing Group, Copyright 2015. (**G**) 3D printed instrumented cardiac microphysiological devices for on-line monitoring, reprinted from [38] with permission of Nature Publishing Group, Copyright 2016.

Several pioneering research works showed that synthetic scaffold exhibited good biocompatibility and facilitated cell growth in vitro [52,93,94]. By employing melt-based EHDP technique, Hochleitner et al. [95] successfully stacked sub-micron filaments (817 ± 165 nm) upon each other of 50 layers to produce a box scaffold (100.6 ± 5.1 μm) with uniform morphology. In the subsequent cell experiments, it was observed that human mesenchymal stromal cells (hMSCs) adhesion was directed to the scaffold rather than attached to the substrate. After 10 days culture, the hMSCs were able to bridge across and then fill the box pores. Bas et al. [96] fabricated 3D structures consisting of Polycaprolactone (PCL) scaffolds infiltrated with hydrogels. They found that varied porosities and structures of the electrohydrodynamically printed scaffolds would lead to different intensions of the hydrogels. Visser et al. [97] embedded human chondrocytes in reinforced gelatin methacrylamide (GelMA) hydrogels, and demonstrated that chondrocytes retained their spherical morphology after 7-day culture. Moreover, they compared the strength of the reinforced hydrogels with native cartilages, and the results indicated that the reinforced hydrogels owned similar stress-strain curves as the healthy native cartilages. The 3D printed PCL scaffold, hydrogel and cell culture are shown in Figure 7A–F.

Our group [52,93,98] had successfully employed both melt-based and solution-based EHDP techniques to fabricate tissue engineering scaffolds with complex curved geometries and microscale fibrous structures, which exhibited good biocompatibility and facilitated cell alignment and proliferation in vitro. Hydroxyapatite nanoparticles were also incorporated into PCL to obtain composite scaffolds for bone tissue engineering, mimicking the collagen fibers and Hydroxylapatite (HA) crystals in natural bone.

3D printing is also broadly used as a microfabrication technology to generate perfusable, vascularized scaffolds [99]. Kolesky et al. [35] employed DIW to fabricate thick 3D cell-laden tissues with embedded vasculature via printing of sacrificial materials. Lee et al. [100] developed a 3D printing method to construct 3D hydrogel with both large fluidic vascular channels (lumen size of ~1 mm) and adjacent capillary network. Skylar-Scott et al. [20] used multimaterial TPP to generate scaffold with high-resolution microcapillary networks and patterned protein cues simultaneously.

### 3.5. Organs-on-Chips

Organs-on-chips are microfluidic devices for culturing microengineered tissues within continuously-perfused microbioreactor to mimic physiological functions of tissues and facilitate screening of new therapeutics such as liver, heart, and skin [35,36]. Previously, the microfluidic systems are mainly prepared using conventional microfabrication methods, such as soft lithography, replica molding, and microcontact printing technique [101,102]. However, these methods not only require expensive microfabrication devices during the fabrication process, but also have difficulties creating complex and organized 3D microscale structures composed of various cell types, extracellular matrix (ECM), and many other elements. The advent of 3D printing has shown great promise to overcome all these challenges [103,104].

With a combination of 3D printing microfluidic devices and tissues in a single-step fabrication process, organs-on-chips would be far more cost-effective and productive, and thus has emerged as a promising alternative to animal studies and conventional cell cultures for biomedical research [32,105]. 3D printing method was easier and more versatile for the production of organs-on-chips with precise controlled functional detection [106,107,108]. It was demonstrated that protein absorption on the printed platform was very low, leading to an accurate measurement of metabolism and drug sensitivity, compared with the traditional 3D cells or tissues models. Anderson et al. [109] fabricated a fluidic chip that contained eight channels in parallel for cell culture. They integrated porous membrane on channel top to demonstrate the potential for high-throughput reading in molecular transport and drug test studies. Moreover, multiple cell types, biomaterials and other components of the microfluidic device were successfully printed with precise and reproducible spatial control for various organ-on-a-chip applications, which would promote full mimicry of the natural conditions of the organs [110]. With exception to reduce uses of harsh chemicals and decreases lengthy fabrication time, integrated solid freeform fabrication system with four print heads (a photopolymer head, a UV head, a microplasma head, and a biologics head) was fabricated and utilized to investigate cancer cells in a co-cultured microfluidic environment. 

Recently, Lind et al. [38] developed a new class of cardiac microphysiological chips embedded with soft sensors through multimaterial 3D printing, to provide non-invasive, continuous electronic readout of the contractile stress of multiple laminar cardiac micro-tissues, as shown in Figure 7E. Specifically, they designed six functional inks based on piezo-resistive, high-conductance, and bioavailable soft materials and printed them at the microscale in a single continuous procedure to characterize micro-architectures that guided the self-assembly of physio-mimetic laminar cardiac tissues. Compared with the previous biomimetic microsystems which were not well suitable for higher-throughput or longer-term studies [111,112], the novel system of 3D printed instrumented cardiac microphysiological devices would drastically simplify data acquisition and leverage the ability to track the temporal development in tissue mechanics, enabling new insights into tissue development and drug-induced structural and functional remodeling.

## 4. Conclusions and Future Perspectives

The field of 3D printing has progressed substantially since its introduction, with applications spreading across multiple fields. In this review, we have presented state-of-the-art high-resolution 3D printing techniques as well as their typical applications in the research of metamaterials, energy storage, flexible electronics, tissue engineering scaffolds and organ-on-chips, as shown in Table 1. A wide variety of materials could be printed precisely and programmably to generate functional 3D objects with predesigned micro/nanoscale structures and compositional gradients. However, their applications in industry are still challenging owing to their limited production efficiency, high costs, and inconstancy. For example, compared with traditional subtractive manufacturing approaches, the low speed of layer-by-layer process still makes it difficult for additive manufacturing technologies to satisfy the demands of mass production in the industries. High cost is another great problem that needs to be solved. Many high-resolution 3D printing techniques can only be utilized in scientific research, especially for TPP which need very expensive proprietary equipment and consumable materials. There is a great potential market for high-resolution 3D printing technologies in the areas of tissue engineering and biomedical engineering. However, existing 3D printing techniques can only fabricate simple tissues with limited functions such as skin. The huge demand of vital organs such as heart, liver and kidney still cannot be satisfied, while there is still a long way to go for the practical clinical applications. 

Although high-resolution 3D printing techniques are still in the early stage of development, confronted with challenges, they show great potential to fabricate multifunctional materials. More research works are being done for the development of new modeling and design methods, materials, equipment, techniques, and even new working principles [114]. For example, continuous liquid interface production was proposed as a novel 3D printing method with a high print speed, which allows parts to be produced in minutes instead of hours [115]. This will lead to better high-resolution 3D printing systems for fabricating micro/nanoscale architecture for practical applications.

## Figures and Tables

**Table 1:** Structure resolution, material selection and applications of high-resolution 3D printing.

Method	Precision	Typical Materials	Applications	Reference
PµSL	5 μm	Photo-active polymer	Metamaterials fabrication, micromatrices to simulate biological systems, MEMS	[22,23,28]
TPP	40 nm	Photo-active polymer	Metamaterials fabrication, MEMS	[4,11,12,13,16]
EHDP	30 nm	PLA, PCL, PEO, particles	MEMS, biosensor, tissue engineering, flexible electronics	[21,46,51]
DIW	1 μm	Hydrogel, silicone elastomer, metal, wax	Flexible electronics, biosensor, energy storage, biomedicine	[30,33,35,38,83,113]
